# Accumulate evidence for IP-10 in diagnosing pulmonary tuberculosis

**DOI:** 10.1186/s12879-019-4466-5

**Published:** 2019-10-30

**Authors:** Xia Qiu, Tao Xiong, Xiaojuan Su, Yi Qu, Long Ge, Yan Yue, Yan Zeng, Wenxing Li, Peng Hu, Dezhi Mu

**Affiliations:** 1 Department of Pediatrics, West China Second University Hospital, Sichuan University, Chengdu, 610041 Sichuan China; 2 Key Laboratory of Obstetric & Gynecologic and Pediatric Diseases and Birth Defects of Ministry of Education, Sichuan University, Chengdu, China; 3 Evidence Based Social Science Research Center, School of Public Health, Lanzhou University, Lanzhou, China

**Keywords:** Pulmonary tuberculosis, Interferon gamma-induced protein 10, Diagnosis, Meta-analysis

## Abstract

**Backgrounds:**

Pulmonary tuberculosis (PTB) is a major health and economic burden. Accurate PTB detection is an important step to eliminating TB globally. Interferon gamma-induced protein 10 (IP-10) has been reported as a potential diagnostic marker for PTB since 2007. In this study, a meta-analysis approach was used to assess diagnostic value of IP-10 for PTB.

**Methods:**

Web of Science, PubMed, the Cochrane Library, and Embase databases were searched for studies published in English up to February 2019. The pooled sensitivity, specificity, positive likelihood ratio (PLR), negative likelihood ratio (NLR), diagnostic odds ratio (DOR), the area under the curve (AUC) and hierarchical summary receiver operating characteristic (HSROC) curve were estimated by the HSROC model and random effect model.

**Results:**

Eighteen studies including 2836 total participants met our inclusion criteria. The pooled sensitivity, specificity, PLR, and NLR of IP-10 for PTB detection were 86, 88%, 7.00, and 0.16, respectively. The pooled DOR was 43.01, indicating a very powerful discriminatory ability of IP-10. The AUC was 0.93 (95% CI: 0.91–0.95), showed the accuracy of IP-10 was good. Meta-regression showed that there was no heterogeneity with respect to TB burden, study design type, age, IP-10 assay method, IP-10 condition and HIV-infection status.

**Conclusions:**

Our results showed that IP-10 is a promising marker for differentiating PTB from non-TB.

## Background

Tuberculosis (TB), a highly contagious disease, is still a major health and economic burden [1]. Globally, approximately 10 million individuals developed TB and more than 1.3 million died of the disease in 2017, according to a WHO report [2]. Pulmonary tuberculosis (PTB), accounting for 75% of all TB cases, contributes substantially to TB mortality, especially with HIV co-infection [3, 4]. Correctly discriminating PTB is an important step to eliminate TB by 2030, a goal established by the WHO [2].

In clinical practice, sputum smear microscopy is ineffective for detecting PTB [5]. Specimen culture for *Mtb* provides the most accurate diagnosis [6]. However, the results of microbiological examination and acid-fast bacillus stains depend on the sputum sample. Immunological tests, such as the tuberculin skin test (TST) and interferon-gamma release assay (IGRA), are auxiliary diagnostic tools for PTB [7]. TST has a low specificity in Bacilli Calmette Guerin (BCG)-vaccinated individuals [7]. In children, IGRAs can yield many indeterminate results [8, 9]. Considering these limitations, additional valid tools are required to improve the diagnosis of PTB.

Interferon gamma-induced protein 10 (IP-10), an IFN-gamma-inducible chemokine, could be expressed at 100-fold higher levels than those of IFN-gamma after TB infection [10, 11]. Age and gender do not affect the level of IP-10 [11, 12]. Since 2007, IP-10 has been reported as a potential parameter for PTB detection [7, 13–29].

Many studies have evaluated the diagnostic potential of IP-10 for PTB, but the results are variable. Therefore, the aim of this study was to synthesize and analyze the diagnostic value of IP-10 for PTB.

## Methods

### Literature search

This study followed the Preferred Reporting Items for Systematic Reviews and Meta-Analyses Diagnostic Test Accuracy criteria 2018 (PRISMA-DTA 2018) [30]. The Web of Science, PubMed, the Cochrane Library and Embase databases were used to search for relevant English language citations published up to February 2019. Our search terms were “tuberculosis,” “pulmonary tuberculosis,” “Chemokine CXCL10,” and “interferon gamma-induced protein 10.” Comprehensive literature search strategies were used based on the following combination of MeSH terms, title/abstracts and all fields for these databases (Additional file 1: Table S1). Additionally, the reference lists of the applicable studies, relevant research letters, and reviews were manually searched to find other potentially relevant studies.

### Literature selection

Two investigators independently determined literature eligibility. Studies reporting IP-10 levels for the detection of PTB were included according to the following criteria: (1) reporting on individuals with PTB and non-TB (population); (2) provision of IP-10 in whole blood and plasma as index test; (4) *Mtb* culture as a gold standard, and other reference standard including pathological examination, microscopy and genexpert MTB/RIF test (WHO recommended) [2]; (5) the primary outcomes including diagnostic performance of IP-10 (sensitivity and specificity); (5) randomized controlled trails, prospective and retrospective studies included (study design); (6) more than 10 individuals reported meeting the inclusion criteria. Studies not published in English, other letters (except research letters), conference abstracts, veterinary experiments, reviews and case reports were excluded.

### Data extraction

The following data were extracted: the first author, year of publication, country, TB high-burden, study design, age, number of participants (patients with PTB and non-TB subjects), TB site, non-TB status, cut-off for index test (IP-10), diagnostic reference standard, method and condition for the IP-10 assay, HIV-infection status, sensitivity, specificity, true positive (TP), false positive (FP), false negative (FN), and true negative (TN) for IP-10. Two investigators independently extracted data from eligible articles, and disagreements were resolved by discussing and reaching a consensus.

### Quality assessment

According to the Cochrane Collaboration, two investigators independently reviewed the methodological quality of eligible articles by Quality Assessment of Diagnostic Accuracy Studies tool-2 (QUADAS-2) [31, 32]. Disagreements were resolved by consensus. Revman (version 5.3) was used to perform the quality assessment.

### Data analysis

Excel was used to construct a two-by-two table, including TP, FP, FN, and TN for patients with PTB. Stata (version 14.0) was used to perform the data analysis. The index test had different optimal cut-offs. According to the recommendation of Cochrane Collaboration, the hierarchical summary receiver operating characteristic (HSROC) model by Rutter et al. was utilized when the index test was assessed by applying various thresholds [32, 33]. The HSROC curve was computed with the “metandi” command [34]. Prediction region presented possible point of sensitivity and specificity in the HSROC curve. The summary point showed the pooled sensitivity and specificity under the optimal threshold value. Confidence region reflected the possible summary point.

The main outcomes were the diagnostic performance of IP-10 for detecting PTB by the random effect model, as evaluated by the summary estimates of sensitivity, specificity, positive likelihood ratio (PLR), negative likelihood ratio (NLR), diagnostic odds ratio (DOR), and the area under the curve (AUC). Sensitivity, reflecting the ability of index test to detect patients, calculated by “Sensitivity = TP/(TP + FN)”. Specificity, reflecting the ability of index test to eliminate disease-free, calculated by “Specificity = TN/(FP + TN)”. PLR, a measure of index test for detection potential for disease, could be calculated by the formula “PLR = Sensitivity/(1-Specificity)”. NLR, a measure of index test for detection potential for non-disease, could be calculated by the formula “NLR = (1-Sensitivity)/Specificity”. DOR, a measure for overall accuracy of index test, could be calculated by the formula “DOR = (TP/FN)/(FP/TN)”. AUC, indicated how the index test was accurate, especially exceeded 0.90. 95% confidence interval (CI) was calculated by wilson method and no correction factor applied.

The *I*^2^ value was not suitable for the quantification of heterogeneity in accuracy studies [35]. Thus, to explore potential sources of heterogeneity, we used a meta-regression analysis with the “midas” command. The intercept was zero. Seven subgroups were created: TB high-burden country (yes or no), study design type (cohort or not), age (adults or not), IP-10 method (multiplex cytokines assay or ELISA), IP-10 condition (unstimulated or stimulated), and HIV-infection status (yes/some or no).

The Deeks test was used to assess publication bias using the “midas” command [36]. No publication bias existed when studies evenly distributed on the sides of regression line or *P* value exceeded 0.05 in Deeks’ funnel plot.

The whole process of data analysis was described in Additional file 2.

## Results

### Search results

**Fig. 1:**
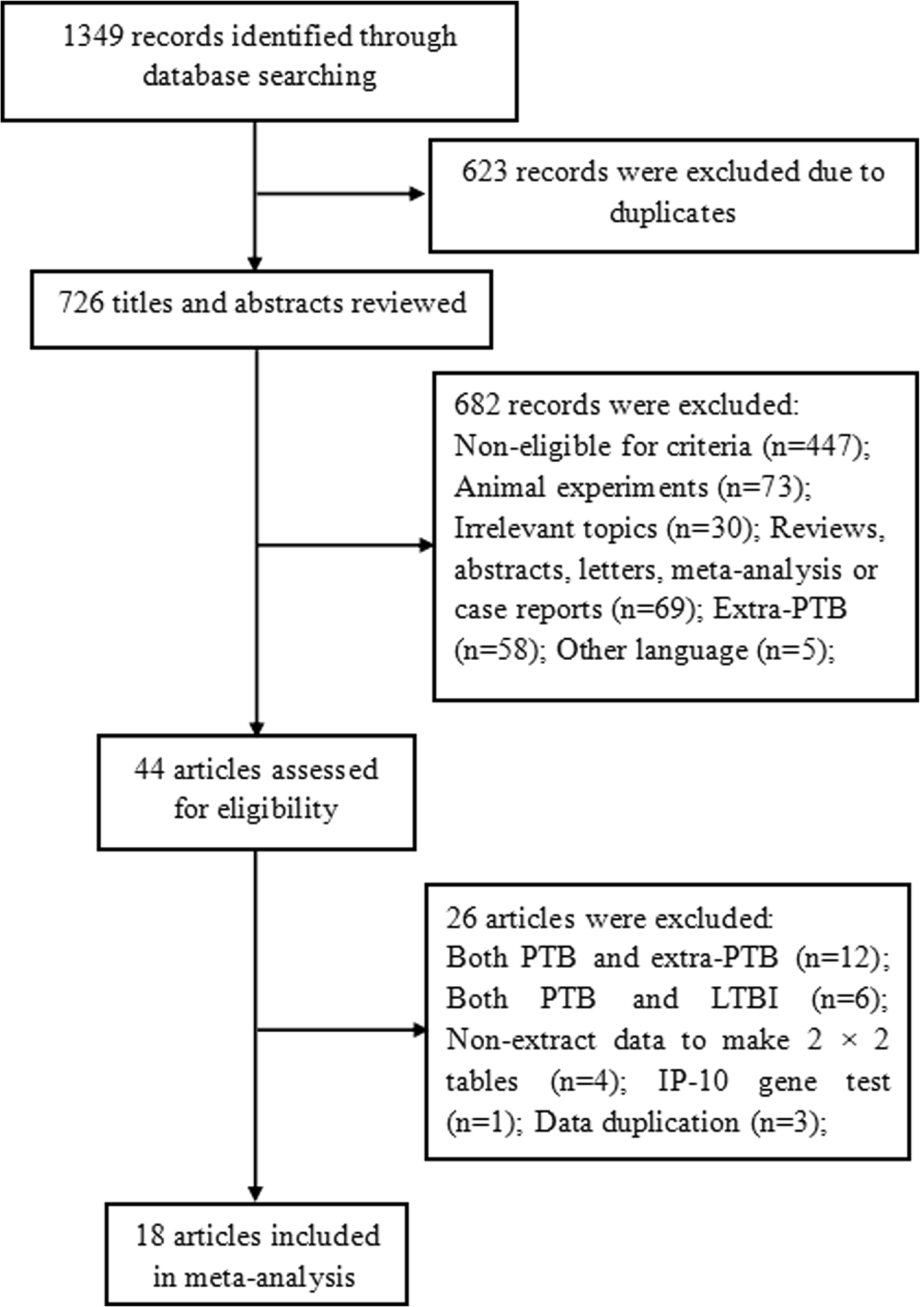
Flow chart of the process of the search strategy for study selection

In total, 1349 records were identified from our literature searches (Fig. 1). After removing 623 duplicates, we read titles and abstracts and excluded 682 records. An additional 447 records were non-eligible for various reasons (e.g., studies involving leprosy, Crohn’s disease, pneumonia, monocyte chemotactic protein-1, interleukin-12, and interleukin-18), 73 records were animal experiments (mouse, calves, warthogs, etc.), 69 records were reviews, abstracts, and letters, 58 records focused on extra-PTB (pleural TB, TB meningitis, osteoarticular TB, etc.), and 5 records were non-English (Chinese, Russian, Polish, etc.). Then, we reviewed the full texts of 44 articles. Ultimately, 18 articles were included in this study.

### Characteristics of included studies

The main characteristics of the 18 articles, comprising 24 trials, are listed in Tables 1 and 2 [7, 13–29]. In total, 2836 participants were involved. The year of publication ranged from 2012 to 2018. Nine (50%) studies were from TB high-burden countries (China, South Africa, India, Thailand, and Uganda), and nine (50%) studies were from TB low-burden countries, according to WHO [2]. Study design, TB site, non-TB status, IP-10 cut-off, and reference standards are summarized in Table 1. IP-10 method, condition, HIV-infection status, cut-off values, sensitivity, specificity, TP, FP, FN, and TN of IP-10 for each trial are shown in Table 2.

**Table 1:** The main characteristics of included studies

Author	Year	Country	TB high-burden country	Study design	Age	Number of participants	TB site	Non-TB status	IP-10 cut-off	Reference standard
Zhao Y [7]	2018	China	yes	prospective cohort	children and adult	41	PTB	healthy controls	not report	A, B
Zhao Y [7]	2018	China	yes	prospective cohort	children and adult	242	PTB	healthy controls	not report	A, B
Zhao Y [7]	2018	China	yes	prospective cohort	children and adult	174	PTB	healthy controls	not report	A, B
Blauenfeldt T [13]	2016	Denmark	no	prospective cohort	adult	225	PTB	healthy controls	2300 pg/ml	B
Jacobs R [14]	2016	South Africa	yes	prospective cohort	adult	55	PTB	non-TB controls (other respiratory diseases)	> 746.6 pg/ml	B
Lee K [15]	2015	Republic of Korea	no	cross-sectional	adult	457	PTB	healthy controls	68.5 pg/ml	A
Azab NY [16]	2013	Egypt	no	prospective cohort	adult	30	PTB	healthy controls	not report	B
Wang X [17]	2012	China	yes	cross-sectional	adult	334	PTB	healthy controls	589 pg/ml	A, B/C
Hong JY [18]	2012	Republic of Korea	no	cross-sectional	adult	78	PTB	healthy controls	2811/119.5 pg/ml	A, B
Kabeer BSA [19]	2012	India	yes	retrospective case control	adult	277	PTB	healthy controls	not report	A, B
Luo J [20]	2018	China	yes	prospective cohort	adult	100	PTB	healthy controls	631.4 pg/ml	A, B
Manna MPL [21]	2018	Italy	no	retrospective cohort	children and adult	47	PTB	non-TB controls (other non-TB pulmonary infections)	> 6557 pg/ml	A, D
Balcells ME [22]	2018	Chile	no	prospective cohort	children and adult	61	PTB	household contacts (QFT -)	> 42.6 pg/ml	A, B
Nonghanphithak D [23]	2017	Thailand	yes	prospective cohort	adult	87	PTB	healthy controls	6319, 2372, 6964 pg/ml	A, B, D
Biraro IA [24]	2015	Uganda	yes	prospective cohort	children and adult	168	PTB	household contacts (TST and QFT -)	4858 pg/ml, 0.47	A
Wergeland I [25]	2015	Norway	no	prospective cohort	adult	58	PTB	non-TB controls (HIV-infected)	2532 pg/ml	A, B
Petrone L [26]	2015	Uganda	yes	prospective cohort	children	52	PTB	healthy controls	209.1 pg/ml	A, B
Latorre I [27]	2014	Spain	no	retrospective cohort	children	149	PTB	healthy controls	2400 pg/ml	A
Wang S [28]	2012	China	yes	cross-sectional	children and adult	142	PTB	healthy controls	451.3 pg/ml	A, B
Jeong YH [29]	2015	Republic of Korea	no	cross-sectional	adult	59	PTB	healthy controls	0.7906	A, C

*Abbreviations*: *TB* Tuberculosis, *PTB* Pulmonary tuberculosis, *QFT* QuantiFERON-TB gold test, *TST* Tuberculin skin test, *HIV* Human immunodeficiency virus, *IP-10* Interferon gamma-induced protein 10, *A* MTB positive cultures, *B* Sputum smear microscopy and/or acid-fast bacilli (AFB) positive, *C* Pathological examination, *D* Genexpert MTB/RIF

**Table 2:** Baseline data of included studies

Author	IP-10 method	IP-10 condition	HIV-infection	Sensitivity (%)	Specificity (%)	TP	FP	FN	TN
Zhao Y [7]	multiplex cytokines assay	unstimulated	no	90.9	100	30	0	3	8
Zhao Y [7]	two cytokines assay	unstimulated	no	90.14	86.21	192	4	21	25
Zhao Y [7]	two cytokines assay	unstimulated	no	60.45	100	81	0	53	40
Blauenfeldt T [13]	ELISA	stimulated (TB Ag)	some	82	97	53	5	12	155
Jacobs R [14]	multiplex cytokines assay	unstimulated	some	86	73	19	9	3	24
Lee K [15]	ELISA	unstimulated	no	70.1	88.3	141	30	60	226
Azab NY [16]	ELISA	stimulated (TB Ag)	no	100	60	10	8	0	12
Wang X [17]	multiplex cytokines assay	stimulated (TB Ag)	no	86	85.9	153	22	25	134
Hong JY [18]	ELISA	stimulated (TB Ag)	no	97.8	87.5	45	4	1	28
Hong JY [18]	ELISA	unstimulated	no	87.5	90.5	21	2	3	19
Kabeer BSA [19]	ELISA	stimulated (TB Ag)	no	91.4	48	160	52	15	48
Luo J [20]	multiplex cytokine assay	unstimulated	no	86	57.14	43	21	7	28
Manna MPL [21]	multiplex cytokine assay	stimulated (TB Ag-nil)	no	80.77	85	21	3	5	17
Balcells ME [22]	multiplex cytokine assay	stimulated (TB Ag-nil)	no	44.1	88.9	15	3	19	24
Nonghanphithak D [23]	ELISA	stimulated (TB Ag)	no	79.2	87.2	38	5	10	34
Nonghanphithak D [23]	ELISA	unstimulated	no	95.8	94.9	46	2	2	37
Nonghanphithak D [23]	ELISA	stimulated (TB Ag-nil)	no	58.3	97.4	28	1	20	38
Biraro IA [24]	ELISA	stimulated (TB Ag)	some	83.3	84.9	85	10	17	56
Biraro IA [24]	ELISA	stimulated (TB Ag/mitogen ratio)	some	96.7	78.7	99	14	3	52
Wergeland I [25]	multiplex cytokines assay	unstimulated	yes	100	92.3	6	4	0	48
Petrone L [26]	ELISA	unstimulated	no	79	93.9	15	2	4	31
Latorre I [27]	ELISA	stimulated (TB Ag)	no	66.7	76.7	8	32	4	105
Wang S [28]	ELISA	stimulated (TB Ag)	no	89.4	81.6	59	14	7	62
Jeong YH [29]	multiplex cytokines assay	stimulated (TB Ag/mitogen ratio)	no	93.9	100	31	0	2	26

*Abbreviations*: *IP-10* Interferon gamma-induced protein 10, *ELISA* Enzyme-linked immuno sorbent assay, *TB Ag* M. tuberculosis-specific antigens, *TB Ag-nil* MTB-specific antigen stimulated minus unstimulated levels, *TP* True positive, *FP* False positive, *FN* False negative, *TN* True negative

### Quality of included studies

The QUADAS-2 tool reflects the methodological quality of included articles (Additional file 3: Figure S1). Patient selection bias was unclear for five studies; one study used a case-control design [19] and four studies did not report the time and consecutiveness of patient enrolment [17, 21, 23, 27]. Additionally, 50% of studies had unclear bias in index tests; in particular, we could not determine whether the results were interpreted in blind conditions [7, 18, 20, 23–25, 27–29]. One study had high risk of bias in the reference standard, which was clinical PTB by clinical presentation and radiological confirmation [16]. Flow and timing bias were unclear in three studies, in which patients were lost in the analysis [19–21]. The applicability concerns were generally low.

### Summary statistics

**Fig. 2:**
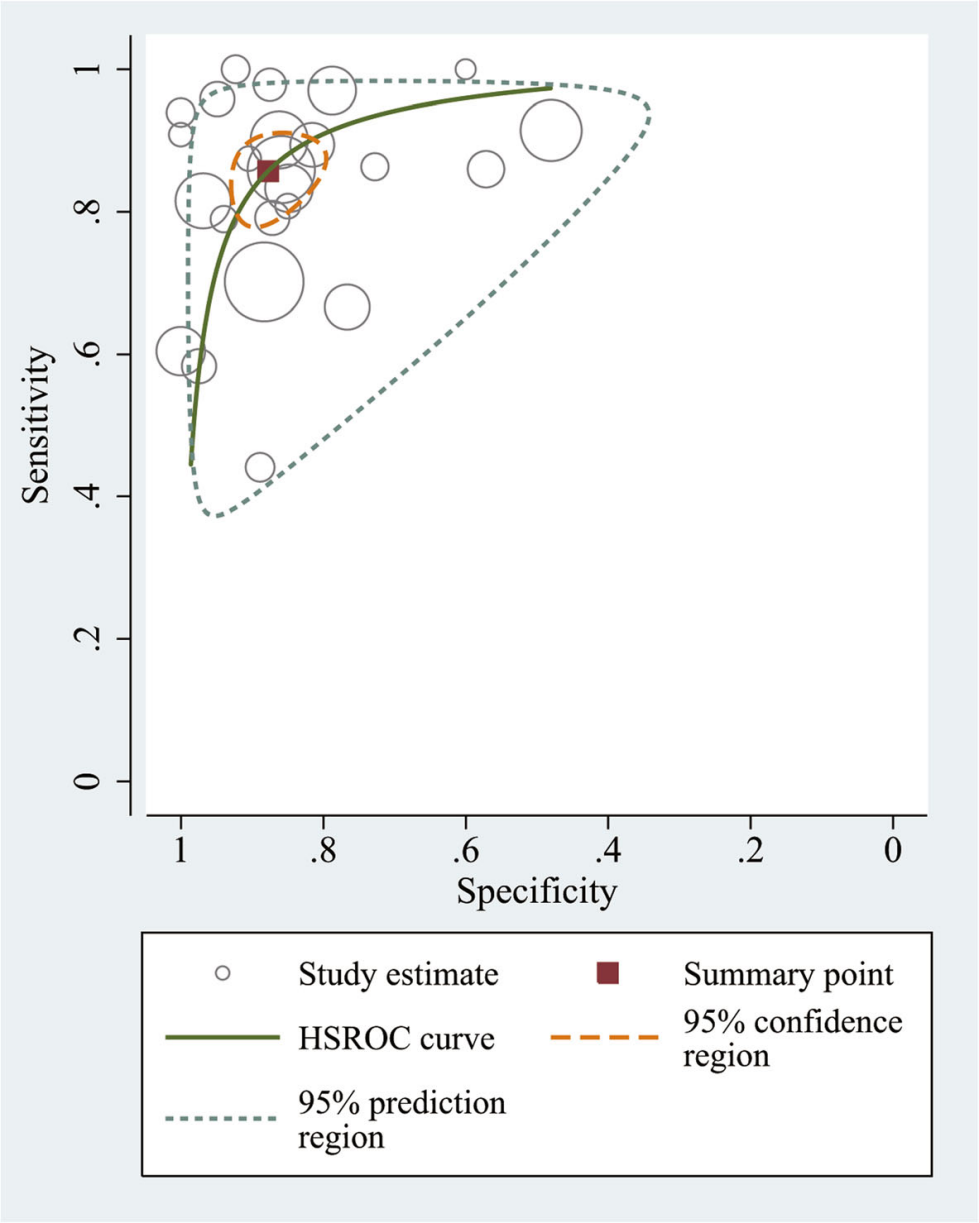
The HSROC curve for assessment of IP-10 for PTB

A total of 2836 participants, comprising 3219 blood samples were included. The sensitivity for IP-10 was 0.86 (95% CI: 0.80–0.90) and the specificity was 0.88 (95% CI: 0.82–0.92). The pooled PLR was 7.00 (95% CI: 4.76–10.30), and the pooled NLR was 0.16 (95% CI: 0.12–0.23). The pooled DOR was 43.01 (95% CI: 25.80–71.69), indicating that the discriminatory effect of IP-10 was good. The AUC was 0.93 (95% CI: 0.91–0.95), showed the accuracy of IP-10 was good. Figure 2 shows the HSROC curves for IP-10, under the optimal threshold value, the pooled sensitivity and specificity were 0.86 and 0.88, respectively.

### Heterogeneity

As shown in Table 3, heterogeneity was assessed by a meta-regression analysis. Heterogeneity was not detected with respect to TB high-burden versus TB low-burden countries (*P* = 0.83), cohort versus other study design types (*P* = 0.55), adults versus children (with or without adults) (*P* = 0.59), multiplex cytokine assay versus ELISA to detect IP-10 (*P* = 0.73), IP-10 stimulation or not (*P* = 0.72), and HIV infection or not (*P* = 0.53).

**Table 3:** Heterogeneity assessment

Covariate	Studies	Sensitivity (95%)	Specificity (95%)	*P* Value (Bivariate Model)
TB high-burden country				
Yes	14	0.86 (0.81–0.92)	0.86 (0.80–0.93)	0.83
No	10	0.85 (0.76–0.93)	0.89 (0.83–0.96)	
Design type				
Cohort	17	0.84 (0.78–0.90)	0.89 (0.83–0.94)	0.55
Cross-sectional/case-control	7	0.89 (0.82–0.96)	0.85 (0.75–0.95)	
Age				
Adults	14	0.88 (0.82–0.94)	0.87 (0.80–0.93)	0.59
Children and adults/children	10	0.83 (0.74–0.91)	0.89 (0.82–0.96)	
IP-10 method				
Multiplex cytokines assay	10	0.84 (0.75–0.92)	0.89 (0.82–0.96)	0.73
ELISA	14	0.87 (0.81–0.93)	0.87 (0.80–0.93)	
IP-10 condition				
Stimulated	14	0.86 (0.80–0.92)	0.86 (0.79–0.93)	0.72
Unstimulated	10	0.86 (0.78–0.93)	0.90 (0.83–0.96)	
HIV-infected				
Yes/some	5	0.90 (0.82–0.98)	0.88 (0.79–0.98)	0.53
No	19	0.84 (0.79–0.90)	0.87 (0.82–0.93)	

### Publication bias

Deeks’ funnel plot showed no statistical significance (*P* = 0.20), indicating no striking publication bias in this study (Additional file 4: Figure S2).

## Discussion

PTB is still a major cause of death worldwide, especially in immunocompromised individuals and children younger than 5 years [37, 38]. The accurate detection and timely treatment of PTB are important components of the “End TB Strategy” globally [39]. Currently, methods for detecting PTB depend on the region, BCG-vaccinated status, HIV status, etc. The search for new markers for the auxiliary diagnosis of PTB is ongoing. Several studies have shown that IP-10 is a promising marker for PTB detection [7, 13–29].

In 2014, Guo et al. published a meta-analysis of studies of IP-10 for diagnosing TB [40]. The diagnostic performance of IP-10 was moderate. In this study, both PTB and extra-PTB individuals were included, and plasma and pleural effusion samples were included. However, the diagnostic standards for PTB and extra-PTB were different. Pleural effusion detection is more traumatic than the use of peripheral venous blood.

Considering these limitations, we performed a meta-analysis to evaluate the overall diagnostic performance of blood IP-10 as a potential biomarker for detecting PTB. We found that IP-10 could be a valuable detection tool (sensitivity: 86%, specificity: 88%). The PLR (7.00>1.00) suggested that IP-10 had good detection potential for PTB. The NLR (0.16<1.00) indicated that IP-10 distinguished non-TB individuals well. The DOR (43.01) indicated a good overall performance of IP-10 in discriminating between PTB and non-TB.

The TST and IGRA, as immunodiagnostic tests, are recommended for the auxiliary diagnosis of PTB by the WHO [2]. The TST could show cross-reactivity in BCG-vaccinated individuals. However, IP-10 is less influenced by BCG vaccination [7]. Ruhwald et al. reported that IP-10 has a much higher sensitivity (92.5%) when compared to the TST (73.9%), and suggested that IP-10 is an alternative biomarker of TST [41]. The recently developed IGRA can overcome some limitations of TST. However, it lacks power when applied to children and individuals coinfected with HIV [9, 14]. IP-10 could be produced at a high level in these populations [42, 43]. Vanini et al. showed that the sensitivity is 66.7% for IP-10-based test and 52.4% for the IGRA in HIV-infected individuals [44].

In bivariate analyses, TB-burden country, study design, age, IP-10 detection method, assay conditions, and HIV infection status were not significant sources of heterogeneity. We also found that the diagnostic performance of IP-10 was similar in multiplex cytokine assays and ELISA (sensitivity: 84% vs. 87%, specificity: 89% vs. 87%). These two methods were comparable with respect to reliability and reproducibility [20]. Considering the cost, ELISA is preferred over multiplex cytokine assays. Stimulated and unstimulated IP-10 had similar diagnostic accuracies for PTB, suggesting that IP-10 could be detected in both conditions. IP-10 had a higher diagnostic potential in HIV-infected individuals, consistent with previous findings [45].

Certainly, our meta-analysis had several limitations. First, we enrolled studies which had various cut-offs of IP-10 assays. In most situations, the investigators of included studies might choose the different cut-offs according to their aims. Second, IP-10 assays are usually performed in combination with conventional tests, but we did not address the reliability and incremental benefit of adding IP-10 to other tests. Third, some studies included patients with PTB after treatment while others did not. Furthermore, the severity and extent of PTB might vary. These factors might influence the diagnostic potential of IP-10. Fourth, heterogeneity could not be ignored. Although the TB-burden country, design type, age, IP-10 method, IP-10 condition and HIV-infection status were not significant sources of heterogeneity in this meta-regression analysis (*P* > 0.05), they could also increase the heterogeneity and reduce the generalizability of the overall performance of IP-10. Furthermore, intercurrent diseases (diabetes mellitus and malignancy) in the included studies might influence heterogeneity.

Despite the low probability of publication bias, it was a concern. Based on the linguistic abilities of our team, only studies written in English were included. The true potential of IP-10 for discriminating PTB from non-TB might be lower than we reported.

## Conclusions

In conclusion, this meta-analysis shows that IP-10 is a promising and reliable marker for differentiating PTB from non-TB. Updated global TB reports should consider IP-10 as an auxiliary diagnostic method for PTB. Furthermore, large, multi-center, prospective studies are warranted to support our findings.

## Supplementary information

**Additional file 1: Table S1.** Pubmed, Web of science, Embase and Cochrane library strategies.

**Additional file 2.** The whole process of data analysis.

**Additional file 3: Figure S1.** Methodological quality of the included articles.

**Additional file 4: Figure S2.** Deeks’ funnel plot the included articles.

## Data Availability

The data generated in this study are included in published article and its supplementary information files.

## Abbreviations

CI: Confidence interval
DOR: Diagnostic odds ratio
FN: False negative
FP: False positive
HSROC: Hierarchical summary receiver operating characteristic
IP-10: Interferon gamma-induced protein 10
NLR: Negative likelihood ratio
PLR: Positive likelihood ratio
PTB: Pulmonary tuberculosis
QUADAS-2: Quality assessment of diagnostic accuracy studies tool-2
TN: True negative
TP: True positive
