# Supplementary material for: Accumulate evidence for IP-10 in diagnosing pulmonary tuberculosis
Source: BMC Infect Dis. 2019 Oct 30;19:924. doi: 10.1186/s12879-019-4466-5 (PMC6822474; doi:10.1186/s12879-019-4466-5)
Supplement: Supplementary file 1 — Additional file 1: Table S1. Pubmed, Web of science, Embase and Cochrane library strategies. [file 12879_2019_4466_MOESM1_ESM.docx]

| Pubmed strategy (308): |
| --- |
| (((((("Tuberculosis"[Mesh]) OR (((((((tuberculosis[Title/Abstract]) OR pulmonary tuberculosis[Title/Abstract]) OR TB[Title/Abstract]) OR PTB[Title/Abstract]) OR mycobacterium tuberculosis Infection[Title/Abstract]) OR tuberculosis infection[Title/Abstract])))))) AND (((((("Chemokine CXCL10"[Mesh]) OR (((((Chemokine CXCL10[Title/Abstract]) OR interferon gamma-induced protein 10[Title/Abstract]) OR interferon-inducible protein 10[Title/Abstract]) OR CXCL10[Title/Abstract]) OR IP-10 [Title/Abstract]))))))) |
| Web of science strategy (534): |
| #1 Topic: (tuberculosis) OR Topic: (pulmonary tuberculosis) OR Topic: (TB) OR Topic: (PTB) OR Topic: (mycobacterium tuberculosis Infection) OR Topic: (tuberculosis infection) #2 Topic: (“Chemokine CXCL10”) OR Topic: (IP-10) OR Topic: (“interferon gamma-induced protein 10”) OR Topic: (“interferon-inducible protein 10”) OR Topic: (CXCL10) #3 #1 AND #2 |
| Embase strategy (492): |
| #1 ‘tuberculosis’/exp/mj OR ‘pulmonary tuberculosis’ OR ‘TB’ OR ‘PTB’ OR ‘mycobacterium tuberculosis Infection’ OR ‘tuberculosis infection’  #2 ‘Chemokine CXCL10’/exp/mj OR‘IP-10’ OR ‘interferon gamma-induced protein 10’ OR ‘interferon-inducible protein 10’ OR ‘CXCL10’  #3 #1 AND #2 |
| Cochrane library strategy (15): |
| #1 MeSH descriptor: [tuberculosis] explode all trees  #2 tuberculosis or pulmonary tuberculosis or TB or PTB or mycobacterium tuberculosis Infection or tuberculosis infection (Word variations have been searched)  #3 #1 or #2  #4 MeSH descriptor: [Chemokine CXCL10] explode all trees  #5 Chemokine CXCL10 or IP-10 or interferon gamma-induced protein 10 or interferon-inducible protein 10 or CXCL10 (Word variations have been searched)  #6 #4 or #5  #7 #3 and #6 |
