# Supplementary material for: Accumulate evidence for IP-10 in diagnosing pulmonary tuberculosis
Source: BMC Infect Dis. 2019 Oct 30;19:924. doi: 10.1186/s12879-019-4466-5 (PMC6822474; doi:10.1186/s12879-019-4466-5)
Supplement: Supplementary file 2 — Additional file 2. The whole process of data analysis. [file 12879_2019_4466_MOESM2_ESM.docx]

The whole process of data analysis was as follows.

First, we installed the two package “ssc install midas” and “ssc install metandi” in STATA, which were the packages of diagnostic meta-analysis. Then, we input the commands “midas tp fp fn tn, res(sum)” and got the values for sensitivity, specificity, positive likelihood ratio, negative likelihood ratio and diagnostic odds ratio. We input the commands “midas tp fp fn tn, plot sroc(both)” and got the value for the area under the curve. We input the commands “metandi tp fp fn tn, plot” and got the hierarchical summary receiver operating characteristic curve. We input the commands “midas tp fp fn tn, reg(burden type age method condition hiv)” for the results of meta-regression. We input the commands “midas tp fp fn tn, pubbias” for publication bias.

Additionally, you could input “help midas” and “help metandi” in STATA 14.0 for more details about our data analysis.
