# Supplementary figures and images for: Accumulate evidence for IP-10 in diagnosing pulmonary tuberculosis
Source: BMC Infect Dis. 2019 Oct 30;19:924. doi: 10.1186/s12879-019-4466-5 (PMC6822474; doi:10.1186/s12879-019-4466-5)

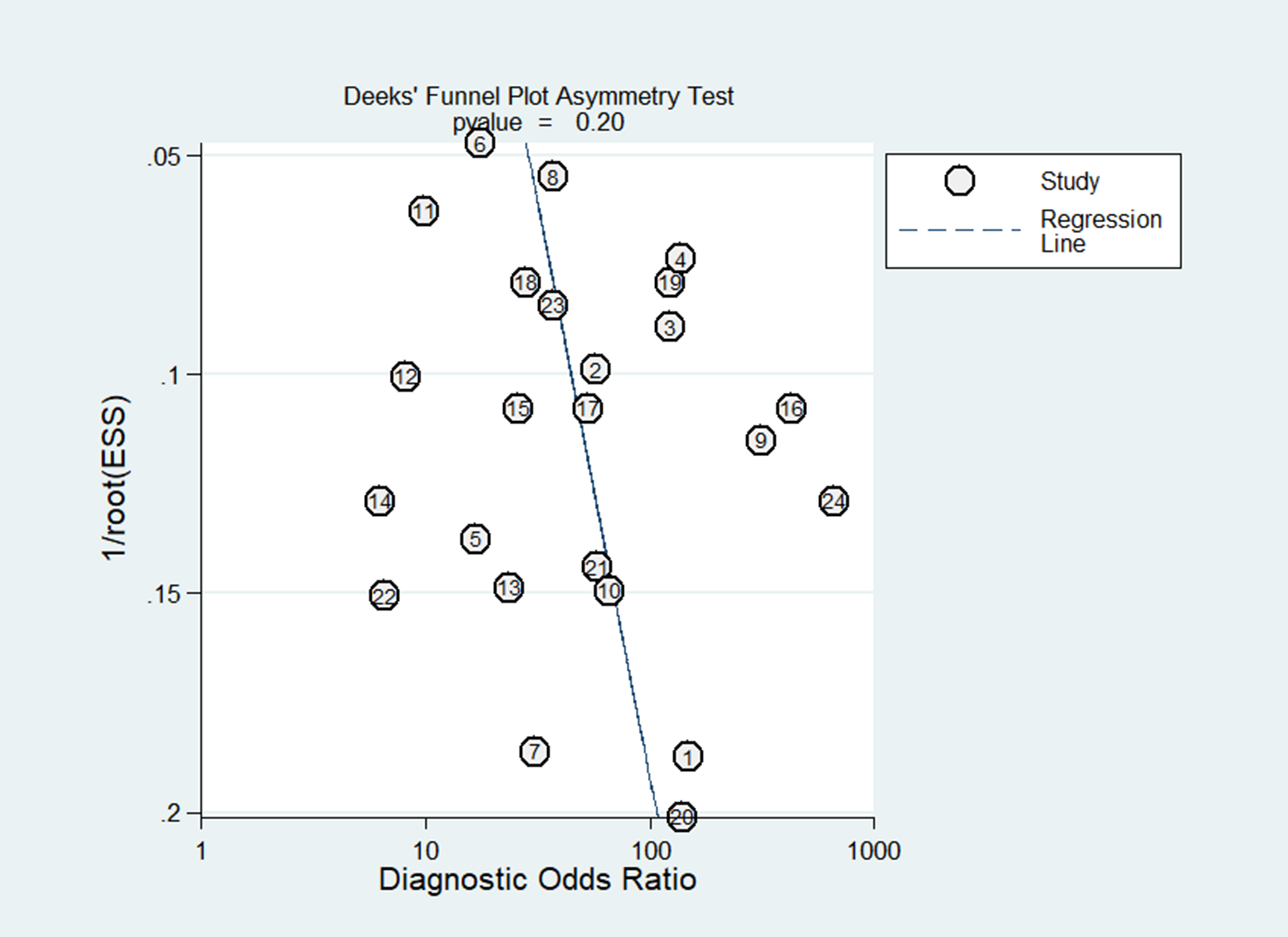

Supplement: Supplementary file 4 — Additional file 4: Figure S2. Deeks’ funnel plot the included articles. [file 12879_2019_4466_MOESM4_ESM.tif]
